# Does intravenous contrast-enhanced computed tomography cause acute kidney injury? Protocol of a systematic review of the evidence

**DOI:** 10.1186/2046-4053-3-94

**Published:** 2014-08-22

**Authors:** Jeanne Françoise Kayibanda, Swapnil Hiremath, Greg A Knoll, Dean Fergusson, Benjamin JW Chow, Wael Shabana, Ayub Akbari

**Affiliations:** 1 Kidney Research Centre, Ottawa Hospital Research Institute, Ottawa, Ontario K1Y 4E9, Canada; 2 Division of Nephrology, Faculty of Medicine, University of Ottawa, Ottawa, Ontario K1N 6N5, Canada; 3 Clinical Epidemiology Program, Ottawa Hospital Research Institute, Ottawa, Ontario K1Y 4E9, Canada; 4 Division of Cardiology, University of Ottawa Heart Institute, Ottawa, Ontario K1Y 4W7, Canada; 5 Department of Medical Imaging, Faculty of Medicine, University of Ottawa, Ottawa, Ontario K1N 6N5, Canada

**Keywords:** Contrast nephropathy, Acute kidney injury, Systematic review, Meta-analysis, Intravenous contrast, Contrast-enhanced computed tomography

## Abstract

**Background:**

Contrast-induced acute kidney injury is a common cause of iatrogenic acute kidney injury (AKI). Most of the published estimates of AKI after contrast use originate from the cardiac catheterization literature despite contrast-enhanced computed tomography (CT) scans being the more common setting for contrast use. This systematic review aims to summarize the current evidence about (1)the risk of AKI following intravenous (IV) contrast-enhanced CT scans and(2) the risk of clinical outcomes (i.e. death, hospitalization and need for renal replacement therapy) due to IV contrast-enhanced CT scans.

**Methods/Design:**

A systematic literature search for published studies will be performed using MEDLINE, EMBASE and The COCHRANE Library databases. Unpublished studies will be identified by searching through grey literature. No language restriction will be applied.

The review will consider all studies that have examined the association between IV contrast media and AKI. To be selected, the study should include two arms: one group of exposed patients who received IV contrast material before CT scans and one group of unexposed group who did not receive contrast material before CT scans. Two authors will independently screen titles and abstracts obtained from electronic databases, extract data and will assess the quality of the studies selected using the Cochrane's ‘Risk of Bias’ assessment tool for randomized trials and the Newcastle-Ottawa Scale for observational studies. A random-effects meta-analysis will be performed if there is no remarkable heterogeneity between studies.

**Discussion:**

This systematic review will provide synthesis of current evidence around the effect of IV contrast material on AKI and other clinical outcomes. Results will be helpful for making evidence-based recommendations and guidelines for clinical and radiologic settings.

**Systematic review registration:**

PROSPERO CRD42013003799.

## Background

Contrast-induced acute kidney injury (CI-AKI) is the third most common cause of acute kidney injury (AKI) and the leading iatrogenic and thus potentially preventable cause of AKI [1]. CI-AKI is associated with long-term adverse outcomes including stroke, myocardial infarction, end-stage renal disease that requires dialysis, and death [2-6]. The average in-hospital cost of contrast-induced nephropathy is estimated at US$10,345 while the 1-year cost of treating a patient with CI-AKI is US$11,812 [7].

Some studies suggest that the risk of AKI is lower after contrast-enhanced computed tomography (CT) scans compared to the risk following arterial cardiac catheterization [8,9]. However, practice guidelines and most clinicians do not differentiate between arterial and intravenous (IV) routes of contrast administration [10,11]. As such, practice guidelines recommend that the same prophylactic measures (e.g. volume expansion) be taken to prevent CI-AKI regardless of whether the contrast is being given intra-arterial (IA) or IV [10-13]. Indeed, data regarding prevention of AKI when contrast is administered by the IV route, such as with a CT scan, is quite limited [9,14]. Further, the evidence indicating the independent effect of IV contrast material on AKI remains uncertain. To the best of our knowledge, only two systematic reviews [15,16] synthesized results from studies on the association between IV contrast administration and CI-AKI. These two reviews included a total of 13 studies (25,950 patients) conducted between 1985 and 2008 and comparing the incidence of AKI between a group of patients who received contrast material and a control group. They concluded no significant difference in the incidence of AKI between contrast patients and control group. This conclusion was confirmed by the meta-analysis performed in one of the two systematic reviews [15] showing a relative risk of developing an AKI after IV contrast material of 0.79 [95% confidence interval (CI) 0.62–1.02, *P* = 0.07] in patients who received intravenous contrast material compared to the control group.

However, on closer inspection, the conclusion of these reviews should be regarded with caution because of the limitations in the methodology of the included studies. In fact, although substantial heterogeneity between patients who underwent contrast material and control group was noted (especially differences in clinical and baseline characteristics), the controls for potential confounders have been completely lacking in most studies. For example, ten studies out of 13 included in these reviews failed to adjust for patient-related confounding factors. Further among studies that performed adjusted analysis [17-19], only two [17,19] have controlled for the most factors indicating the status of renal function, i.e. baseline serum creatinine (SCr) or glomerular filtration rate (GFR) at admission. Additionally, the definition of CI-AKI as well as the time between the initial SCr determination (baseline) and the post-scan SCr determination varied widely between studies. Such limitations of methodology could result in an overestimation or underestimation of the effect of IV contrast material associated to the AKI.

More recently, two studies [20,21] used propensity matching analysis to estimate the effect of IV contrast material on AKI, a statistical technique with which the effects of confounding bias can be reduced in observational studies [22,23]. In fact, this method estimates the probability of subjects for being exposed to an exposure factor according to their baseline characteristics and attributes a score to each subject. Subsequently, subjects in exposed group and those in unexposed group who have a similar score are matched. Thus, the matching of subjects with a similar score mimics a randomization assignment because the distribution of baseline covariates will be the same among exposed and unexposed subjects. In a sample of 20,242 patients, Davenport et al. [20] reported a post-CT AKI odds ratio (OR) of 1.26 (95% CI 1.07–1.49; *P* = 0.007) in patients with pre-CT SCr levels of 1.5 mg/dL or greater, while McDonald et al. [21] reported an OR of 0.97 (95% CI 0.81, 1.16; *P* = 0.76) in 19,651 patients with a baseline SCr of 1.5–2.0 mg/dL and an OR of 0.91 (95% CI 0.66, 1.24; *P* = 0.58) in 12,207 patients with a baseline SCr of 2.0 mg/dL or more.

Given the methodological weaknesses of studies included in the previous systematic reviews and the discrepant results reported by new research that used more robust methods [20,21], an up-to-date systematic review is needed to determine the current evidence around the effect of intravenous contrast material on AKI.

The aims of this systematic review will be to summarize and quantitate (1) the risk of AKI in patients who underwent intravenous contrast-enhanced CT scans compared to patients who did not undergo contrast-enhanced procedures and(2) the risk of clinical outcomes (i.e. death, hospitalization and need for renal replacement therapy) due to intravenous contrast in patients who underwent intravenous contrast-enhanced CT scans compared to patients who did not undergo contrast-enhanced procedures.

## Methods/Design

### Eligibility criteria

#### ***Participant/population***

This review will consider all studies conducted among adult patients aged ≥18 years. Studies must include results of serum creatinine concentration or GFR assessed prior to and after CT scan. Studies in patients who received contrast material by intra-arterial, intracoronary only or both intravenous and intra-arterial routes will be excluded.

#### ***Type of exposure***

The exposure to be considered will be the contrast material administered by IV routes.

#### ***Type of outcomes***

The primary outcome of interest will be AKI following IV contrast-enhanced CT scans. Secondary outcomes will include death, hospitalization and need for renal replacement therapy.

#### ***Type of comparison***

Patients who underwent IV contrast-enhanced CT scans will be compared to patients who did not undergo contrast-enhanced procedures.

#### ***Types of studies***

The review will consider all studies that have examined the effect of IV contrast material on AKI (randomized trials and observational studies). To be selected, a study should include two arms: one group of exposed patients who received IV contrast material before CT scans and one group of unexposed group who did not receive contrast material before CT scans. The study also has to report one of the outcomes of interest mentioned above. Systematic reviews and meta-analysis will be excluded, but the bibliography of any reviews retrieved will be scanned for relevant citations.

### Search strategy

The search strategy will be designed to access both published and unpublished studies.

To identify published studies, we will perform a systematic literature search using electronic bibliographic databases: Medline, Embase and The Cochrane Library.

An adapted search strategy will be used to search all the databases from their start date up to the date the search is run. The search terms will be adapted for the different databases using a combination of Medical Subject Heading (MeSH) and relevant keywords contained in titles and abstracts. An information scientist will help in developing and conducting the search strategy. We will update the literature search until the draft manuscript is submitted for peer review. Articles identified will be downloaded and imported directly in Endnote. Additional studies will be identified by reviewing the bibliographic reference lists of studies selected from electronic databases and from relevant systematic reviews.

To identify unpublished studies, we will search through grey literature including reports and conference abstract books.

To reduce language bias, we will consider articles published in any language.

### Study selection and data extraction

To select studies of interest, at least two authors (from JFK, SH and AA) will independently screen titles and abstracts obtained from electronic databases and will exclude irrelevant articles. A data extraction tool will be developed based on the Cochrane Consumers and Communication Review Group's data template [24].

Pre-specified data elements from each selected study will be recorded including study authors, year of study, year of data collection, country, study design, patient characteristics and setting (e.g. stroke, trauma), data on AKI (definition, pre- and post-scan creatinine concentration, time collection of post-scan creatinine concentration), data on contrast material (type and volume of contrast), prophylactic measures, confounding variables included in the analysis (such as age, sex, comorbidities), sample size in exposed and control patients, measures of primary outcome and secondary outcome (incidence rate, odds ratio, hazard ratio), statistical tests carried out, significance (*P* values), precision (confidence intervals) and other important variables.

JFK and SH will extract and compare the data from the selected studies. Disagreements between JFK and SH will be resolved by consensus. A third author (AA) will review all extracted data. Disagreements between JFK, SH and AA will be resolved by consensus. If consensus cannot be reached between the three reviewers, another investigator (GK) will make the final decision.

### Quality assessment

The goal of this assessment is to make a methodological judgement of whether the design and conduct of the study compromised the validity of the association between exposure (IV contrast material) and the outcome (AKI or other clinical outcomes).

The study quality and the presence of potential bias within studies will be assessed independently by two authors (JFK and SH) using the Cochrane's ‘Risk of Bias’ assessment tool for randomized studies [25] and the Newcastle-Ottawa Scale for observational studies [26].

### Data synthesis and analysis

If there is no heterogeneity according to the quality of methods and bias assessment between studies selected, we will pool results of all comparables studies into a meta-analysis using the Comprehensive Meta-analysis software (version 2.2.046, Biostat Inc., Englewood, NJ, USA). Risk ratios and their 95% CI will be calculated from the data generated by each included study. To test heterogeneity of the effect of IV contrast material on the risk of development of AKI and other clinical outcomes reported by different studies, we will use Cochran's *Q* statistic test (*P* value <0.1 is considered significant) and the *I*^2^ statistic. The overall effects for the outcomes and their 95% confidence intervals will be obtained using a random-effects model as described by DerSimonian and Laird [27].

Sensitivity and multivariate meta-regression analyses will be performed to assess the effects of some clinical factors and methodological quality of studies on the meta-analyses estimates. Sensitivity analyses could be those excluding studies with lower methodological quality or those published only as abstracts, while comorbidities (i.e. baseline kidney function, diabetes) will be used as covariates for meta-regression.

We will perform subgroup analyses using reported data on subgroups of patients with underlying chronic kidney disease. A funnel plot will be used to assess for the presence of publication and other reporting biases, and a visual examination of the funnel plot and the Egger's statistic will be used to test for bias.

If it is not possible to conduct meta-analysis because of the heterogeneity of studies, a narrative analysis will be carried out describing the characteristics and quality of studies included as well as explaining the relationship between IV contrast material and AKI (size, direction and consistency of effect).

## Discussion

In recent years several meta-analyses addressed the question of nephropathy following intravascular contrast material [28-31]. These meta-analyses included both IA and IV studies that were interested either in measuring the incidence and risk factors of CI-AKI in patients undergoing intravascular contrast material [30,31] or in comparing different CI-AKI prevention methods/different types of contrast media (iso-osmolar and low-osmolar contrast) on risk of CI-AKI [28,29]. However, the IV studies included in three [28,29,31] of the four meta-analyses were conducted exclusively in patients who received contrast material. None included a study comparing patients exposed to IV contrast material to control group of patients who did not receive contrast media. Only the meta-analysis published by Moos et al. included four studies [32-35] (out of 42 included in the meta-analysis) that included a control group of patients who were not exposed to IV contrast material. However, it is not possible to estimate the average effect of IV contrast material on CI-AKI from the estimates reported by these four studies because they were pooled together with estimates reported by other studies that have not include a control group of patients.

Thus, none of the above meta-analyses have examined the causal effect of IV contrast material exposure on CI-AKI, while debates and differences of opinions exist on the nephrotoxic potential of contrast material when administered by IV route [36-39]. Moreover, in certain clinical setting (i.e. cardiac angiography), the risk of intra-arterial contrast material to kidney function is extrapolated to intravenous material without consideration of the evidence [16,36].

Until now, only two systematic reviews [15,16] have synthesized evidence from 13 studies conducted between 1985 and 2008 that compared the risk of AKI between a population of patients who received a contrast material by IV and an unexposed population of patients who did not receive the contrast material. They reported a similar risk of AKI between both populations (contrast exposed patients and unexposed patients). However, this conclusion could be limited by the methodological weaknesses used in studies included in these systematic reviews, especially the lack of control of potential confounding factors and a selection bias resulting from the selection of the control group. In particular, two important methodological limits should be cited. First, we noted that in nine out of 13 studies included in the more recent review [15], the mean baseline SCr was higher among unexposed group than in IV-contrast-exposed group, suggesting a selection bias (higher risk patients did not receive contrast). Second, the authors of this systematic review reported having modified the scale assessing the methodological quality. They excluded the question assessing if the outcome of interest was not present at the start of the study, because the researchers did not determine whether patients had preexisting AKI in any studies included in the systematic review. These limitations could have affected the validity of the association between IV contrast and AKI. In fact, on one hand, to attribute the risk of AKI to IV contrast exposure, it supposes the absence of AKI cases in the two groups under study before the exposure. On another hand, the validity of causal relation between IV contrast material and AKI depends upon the comparability of exposed and unexposed patients.

Since 2008 (date on which the last study included in the more recent systematic review was published), several other studies measuring the effect of IV contrast medium on renal function were published [20,21,34,40-47]. Three among them [20,21,41] used propensity matching methods guaranteeing that the group exposed to IV contrast material and unexposed group had similar baseline characteristics and had a same likelihood of being exposed to IV contrast material. Moreover, two studies [20,21] among the three that used propensity matching methods used data collected over 10 years (from 2000 to 2010) and recruited 41,614 patients. Interestingly, these studies have produced inconsistent results: one reported IV contrast material as being positively associated with AKI, while the other reported no significant association between IV contrast and AKI.

We believe that to include these more recent studies in a new systematic review will improve the validity of available evidence on the association between IV contrast and AKI. In particular, we will have much larger sample size and we anticipate that the quality of methodology was improved in these recent studies.

Finally, since it is essential to provide clinicians with the best available evidence on the causal association between IV contrast and AKI, the results of this systematic review will be helpful in making evidence-based recommendations and guidelines for clinical and radiologic settings.

## Abbreviations

CI-AKI: contrast-induced acute kidney injury; CIN: contrast-induced nephropathy; CT: computed tomography; GFR: glomerular filtration rate; IV: intravenous; IA: intra-arterial; MeSH: Medical subject heading; OR: odds ratio.

## Competing interests

The authors declare that they have no competing interests.

## Authors’ contributions

JFK, SH and AA conceived, designed the research and drafted the protocol. AA, GK, DAF, BC and WS critically revised the protocol for scientific content. All authors read and approved the final manuscript.
